# Prevalence of Screening Colonoscopy among the First-Degree Relatives of Patients with Colorectal Cancer and Related Factors

**DOI:** 10.34172/mejdd.2025.412

**Published:** 2025-04-30

**Authors:** Zahra Rastinmaram, Mohammad Hassan Emami, Sayyed Mohammad Reza Hakimian, Alireza Fahim, Hojatollah Rahimi, Fatemeh Maghool

**Affiliations:** ^1^Poursina Hakim Digestive Diseases Research Center, Isfahan University of Medical Sciences, Isfahan, Iran; ^2^Cancer Prevention Research Center, Isfahan University of Medical Sciences, Isfahan, Iran

**Keywords:** Colorectal cancer, First-degree relative, Colonoscopy, Screening

## Abstract

**Background:**

First-degree relatives (FDRs) of patients with colorectal cancer (CRC) possess a higher risk of developing CRC. Colonoscopy is among the most effective screening methods for preventing CRC. This study aimed to assess screening rates among FDRs of patients with CRC and determine obstacles to screening in this population.

**Methods:**

This cross-sectional study was conducted in Isfahan, Iran. A total of 160 asymptomatic FDRs were identified and considered eligible for inclusion in the analysis.

**Results:**

The mean age of FDRs was 50 years, and 65.6% were at high risk for CRC. Only 32.4% underwent screening according to guidelines, and all of them were classified as high-risk. Index patients (IPs) aged under 50 and receiving a recommendation for screening were identified as two main factors associated with guideline-based CRC screening. Among FDRs who did not undergo colonoscopy, 64.4% were unaware of the risk of CRC, and 56.3% lacked knowledge about the procedure.

**Conclusion:**

Urgent implementation of effective interventions and improved education for both healthcare providers and patients on risk-based CRC screening for FDRs is crucial. Further descriptive investigations are needed to identify barriers to CRC screening in this population.

## Introduction

 Colorectal cancer (CRC) is the third most common cancer in the world, and more than 1.2 million new cases and more than 600 000 deaths are reported annually.^1,2^ A family history of CRC is an important risk factor for advanced neoplasia in asymptomatic individuals compared with other known risk factors such as lifestyle and dietary habits.^3,4^

 First-degree relatives (FDRs) of the patient have a higher absolute and relative risk of developing CRC.^5,6^ Individuals with one FDR diagnosed under the age of 55 or two FDRs diagnosed with CRC at any age have a 3- to 6-fold increased risk.^3-6^ The pathophysiology of CRC involves a long progression from the initial manifestation of an adenomatous polyp to the progression of CRC. Preventive efforts are aimed at early detection and removal of precancerous polyps before they turn into CRC.^7^ The start of screening for CRC and their interval are based on personal risk profile. Therefore, timely screening of FDRs stands as a pivotal approach in mitigating the incidence of CRC.^8,9^

 Colonoscopy and removal of precancerous polyps is one of the most efficacious screening modalities to prevent CRC. Early detection and removal of adenoma reduces the risk of CRC by 95%.^10-12^ In FDR of patients with CRC, screening colonoscopy (SC) can cause a 50% reduction in CRC mortality.^8^ Fecal occult blood test (FOBT) and fecal immunochemical test (FIT) are widely employed for CRC screening, given their non-invasive nature, simplicity, and cost-efficiency. Research findings indicate that FOBT screening is associated with a mortality reduction ranging from 15% to 33% in cases of advanced CRC.^13-16^

 Given the heightened risk linked to a familial history of CRC, it is imperative to prioritize screening for this population. Recent evidence underscores the advantages of such screening initiatives. For instance, individuals from hereditary non-polyposis colorectal cancer (HNPCC) families who engage in routine screening programs demonstrate a 62% decrease in CRC incidence and a 65% decline in mortality rates compared with control groups.^17^

 Current guidelines advocate for the initiation of SC among FDRs of patients with CRC at the age of 40. Alternatively, if the index patient (IP) received a diagnosis at or before the age of 60, screening should commence 10 years earlier than the age of diagnosis of the IP.^9,18,19^ However, studies conducted in the United States have revealed that merely 27.8% of FDRs aged between 41 and 75 undergo SC. Similar findings have been documented in other investigations.^11,20^ Notably, the highest adherence rates among FDRs of younger patients (under 56 years of age) have been observed. Nonetheless, these figures decrease significantly to 6-11% when considering adherence to interval colonoscopy.

 Despite widespread recommendations, the utilization of colonoscopy remains suboptimal. FDRs, who are at a higher risk of developing adenomatous polyps and cancer compared with individuals without a family history, exhibit unsatisfactory screening rates.^8,21,22^ Failure to perform colonoscopy is associated with a wide range of detrimental health consequences, including increased risk of CRC, diagnosis of adenoma at advanced stages, and death from CRC. Therefore, there is a great interest in understanding the impediments and facilitators of colonoscopy.^3,8^

 In Iran, like other developing countries, the rate of CRC is increasing, especially in the new generation. Previous studies have shown that early-onset CRC is higher in families with a family history of CRC.^21,23-25^ Unfortunately, the rate of CRC screening in Iran falls considerably below the optimal threshold. Most individuals at elevated risk for CRC have not undergone colonoscopy screening.^4,25^ Limited research has focused on the awareness level and obstacles to screening among high-risk individuals. Moreover, there is only a small body of data concerning the prevalence of actual screening within this subgroup and individuals’ perceptions, knowledge, or concerns regarding CRC screening.^4,21,25,26^ This study aimed to determine the screening rates among FDR of patients with CRC and identify barriers to screening among this population.

## Materials and Methods

 This cross-sectional study was performed on 160 FDRs of patients diagnosed with CRC, whose information has been registered at Poursina Hakim Digestive Diseases Clinic and Research Center, Isfahan, Iran.

 Patients aged 18 years or older with a history of colon and rectal malignancies were considered index patients. IPs were contacted via telephone, the study’s objectives were explained, and upon consent, information was collected on their age at cancer diagnosis, cancer history in first and second-degree relatives, and awareness of cancer risk among their FDRs. Additionally, contact details of FDRs who volunteered were collected upon obtaining consent. Selected FDRs were invited to the clinic to complete a comprehensive checklist while being informed about cancer risks for FDRs. Data collected from FDRs included demographic details, screening tests (such as colonoscopy, FOBT, FIT), and awareness of cancer risks.

**Figure 1:**
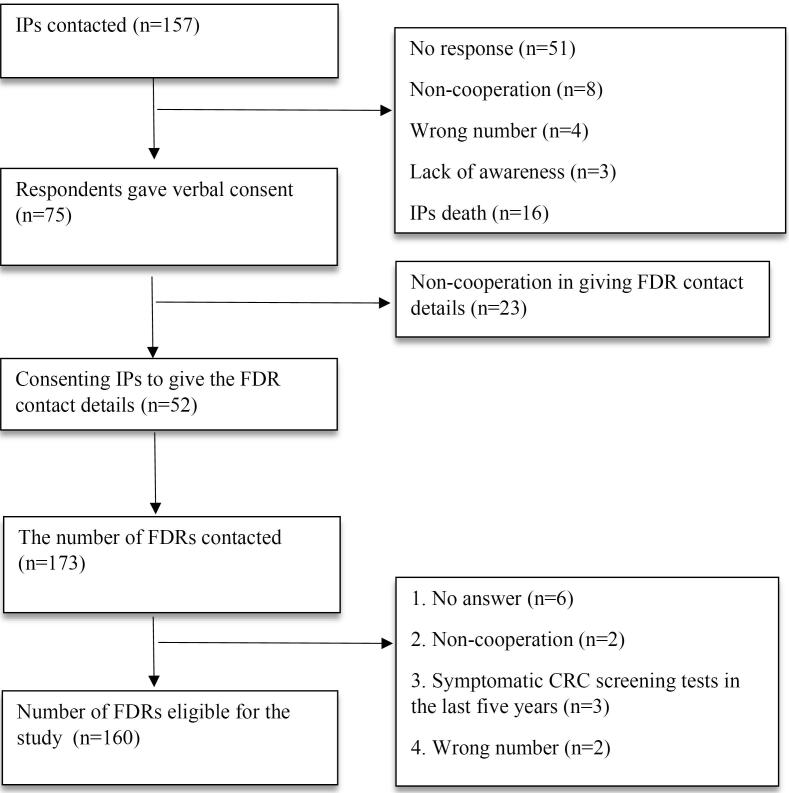
Flowchart of the requirement of asymptomatic first-degree relatives of patients with colorectal cancer. FDRs, First-degree relatives

 Inclusion criteria for FDRs were: being 40 years old or 10 years younger than the age of CRC diagnosis in their family (whichever was earlier), with no history of advanced adenoma, CRC, inflammatory bowel disease (including Crohn’s disease, and ulcerative colitis), or familial adenomatous polyps (FAP). Exclusion criteria included failure to respond to calls, lack of eligibility, or residence in distant cities or countries. If they did not perform sigmoidoscopy, colonoscopy, or FIT tests in the last 5 years, they were classified as asymptomatic and eligible for CRC screening. If any of the screening methods were performed, the reason was asked to make sure that they were asymptomatic at the time of the screening. To minimize bias, strict inclusion and exclusion criteria were applied, standardized data collection methods were used, and informed consent was obtained from all participants to ensure consistency and reduce selection bias. The protocol for the recruitment of FDRs is shown in the Figure 1.

 Risk stratification was based on the screening guideline^8,9^:

1. High risk: FDRs with a first-degree relative diagnosed with CRC or adenomatous polyps before age 60, or having two or more FDRs with these conditions at any age, or exhibiting potential CRC symptoms (i.e., rectal bleeding, changes in bowel habits). For these individuals, colonoscopy was recommended every 5 years starting at age 40 or 10 years before the youngest family member’s diagnosis. For hereditary colon cancer cases (excluding FAP), colonoscopy was advised every two years starting in the third decade of life.
2. Moderate risk: FDRs with a first-degree relative diagnosed with CRC or an adenomatous polyp at age 60 or older or having two second-degree relatives with these conditions. For them, colonoscopy was recommended to begin at age 50 and be repeated every 10 years, and annual FIT testing starting at age 40.

Data were recorded in a specially designed checklist, capturing FDR information such as demographics, screening necessity, screening tests undertaken, sources of screening information, and reasons for not undergoing screening.

 To determine the required sample size at a significance level of 0.05, and assuming the CRC screening rate among FDRs of patients with CRC is 36%, based on the study by Adakan et al (23), the sample size was calculated using the following formula with a precision of 0.08. The resulting sample size was estimated at 146, and accounting for potential dropout, 160 participants were targeted.


$$n=\frac{\left(p*q\right)*z_{1-\alpha /2}^{2}}{d^{2}}$$

###  Statistical Analysis

 Quantitative variables were reported as the mean and standard deviation, and numbers and percentages were applied to the qualitative variables. Student’s *t* test and chi-square/Fisher test were used for continuous and categorical variables, respectively. Simple associations of each independent categorical variable with the CRC screening status were examined, and variables with *P* values less than 0.25 were selected as independent variables in a multivariable logistic regression. *P* < 0.05 was considered significant. All analyses were done using R software (version 4.2.3).

## Results

 Upon contacting the 157 registered IPs, 52 had at least one living FDR. Of the 173 approached FDRs, 92.5% consented to participate (Figure 1). Following interviews and questionnaire administration, 160 asymptomatic and eligible FDRs were identified to be included in the study (Figure 1).

###  Characteristics of IPs

 The mean age of diagnosis CRC in IPs (n = 75, 52% male) was 58 years, with a standard deviation (SD) of 13.8 years. 48% of these patients had a family history of various malignancies, 14.6% reported more than one type of carcinoma in their family, and 18.6% had a history of CRC (Table 1).

**Table 1:** Characteristics of index patients

**Characteristics**	**No.**	**(%)**
Sex		
Male	39	52
Female	36	48
Family history of malignancy		
Without history	39	52
More than one type	11	14.6
Colorectal cancer	14	18.66
Gastric cancer	3	4
Breast cancer	2	2.66
Leukemia	2	2.66
Lung cancer	1	1.3
Brain cancer	1	1.3
Uterus cancer	1	1.3
Biliary tract	1	1.3
	**Mean**	**SD**
Age (y)	60.13	13
Age at diagnosis (y)	57.98	13.8

###  Characteristics of FDRs

 The mean age of FDRs (n = 160, 53% female) of patients with CRC was 50 years, with a standard deviation of 11.3 years. A majority, 65.6%, were at high risk for CRC. 47% were the siblings, 45% offspring, and the minority (8%) were the parents of IPs (Table 2).

**Table 2:** Characteristics of first-degree relatives of patients with colorectal cancer

**Characteristics**	**No.**	**(%)**
Sex		
Male	75	(46.9)
Female	85	(53.1)
Education		
Elementary/no educated	26	(16.3)
High school	19	(11.9)
Diploma and associated degree	65	(40.6)
Bachelor of Science and higher	50	(31.3)
Marital status		
Married	146	(91.3)
Single	14	(8.8)
Employment situation		
Operative	66	(41.3)
Retired	93	(58.1)
Relationship to index case		
Parent	13	(8.1)
Sibling	75	(46.9)
Child	72	(45)
Risk		
Average risk	55	(34.4)
High risk	105	(65.6)
Recommend screening		
No	34	(21.3)
Yes	126	(78.8)
Ever receiving CRC screening		
Average risk	35	(63.6)
High risk	62	(59)
Ever receiving screening colonoscopy		
Average risk	17	(31)
High risk	44	(42)
	**Mean**	**SD**
Age (years)	50	11.3

Note: CRC, Colorectal cancer.

###  FDRs Ever Received Screening Colonoscopy

 Of a total of 160 participant FDRs, 97 (60.6%) underwent screening tests (including FIT and colonoscopy) for CRC (Table 2). 38% of FDRs have undergone colonoscopy screening, and 42% of high-risk patients have undergone colonoscopy screening. Among the FDRs, 50.7% of siblings, 25% of children, and 38.5% of parents of the IPs underwent SC (Table 3). The age group evaluation of those who completed SC showed that 34% of FDRs were under 50 years, 44.7% were between 50 and 59 years, and 42% were equal to or over 60 years. Notably, regarding IP ages, colonoscopy was more frequently conducted among FDRs of IPs aged under 50 years (Table 3). 35.6% of FDRs who underwent CRC screening had primary and high school education, 44.6% had diploma and associate degree degrees, and 32% had bachelor’s degrees and higher. However, there was no significant difference in screening tests across different educational levels (Table 3). Most FDRs (62.5%) had been informed of CRC screening tests, primarily by gastroenterologists (63%), followed by family members (17%), and the minority by public media sources like the Internet and television (data are not shown).

**Table 3:** Characteristics of FDRs of CRC patients receiving screening colonoscopy

**Independent variable**	**All (n=160)**	**Ever had a screening colonoscopy**	**Never had a screening colonoscopy**	***P*** ** value**
Age (y)				
< 50	91 (57)	31 (34)	60 (66)	0.46
50-59	38 (23.8)	17 (44.7)	21 (55.3)	
> 60	31 (19.4)	13 (42)	18 (58)	
IP age (y)				
< 50	45 (28)	25 (**55.6**)	20 (44.4)	
50-59	34 (21.3)	12 (35.3)	22 (64.7)	0.01*
> 60	81 (50.6)	24 (29.6)	57 (70.4)	
Sex				
Female	85 (53)	30 (35.3)	55 (64.7)	0.4
Male	75 (47)	31 (41.3)	44 (58.7)	
Marital status				
Single/widow	14 (8.8)	4 (**28.6**)	10 (71.4)	0.44
Married	146 (91.2)	57 (39)	89 (61)	
Job				
Operative	67 (42)	20 (30)	47 (70)	0.07
Retired	93 (58)	41 (44)	52 (56)	
Relationship to the index case
Sibling	75 (47)	38 (50.7)	37 (49.3)	0.006^*^
Child	72 (45)	18 (25)	54 (75)	
Parents	13 (8)	5 (38.5)	8 (61.5)	
Education				
Primary/High school	45 (28)	16 (**35.6**)	29 (**64.4**)	
Diploma/associated degree	65 (40.6)	29 (**44.6**)	36 (55.4)	0.35
Bachelor and higher	50 (31.3)	16 (32)	34 (68)	
Risk				
Average risk	55 (34.4)	17 (31)	38 (69)	0.17
High risk	105 (65.6)	44 (42)	61 (58)	

*Note*: FDRs, first-degree relatives; CRC, Colorectal cancer; IP, Index patient. * *P* < 0.05.

**Figure 2:**
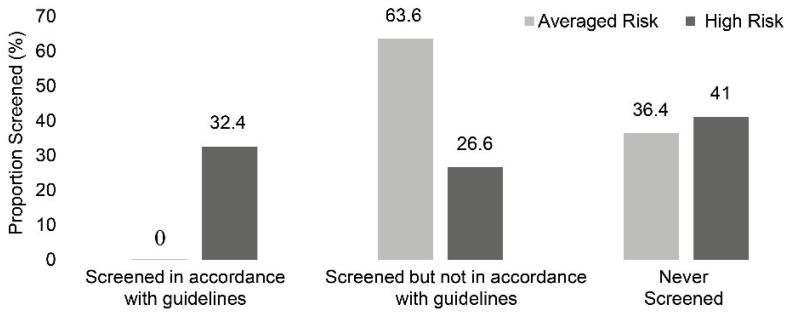
Colorectal cancer screening status in first-degree relatives of patients with colorectal cancer

 Among FDRs who had undergone CRC screening, 42% of high-risk FDRs underwent colonoscopy compared with 31% of average-risk individuals. FIT performance was 36.2% in the high-risk group and 51% in the average-risk group (data are not shown). Out of 160 FDRs, only 32.4% underwent screening under guidelines (Figure 2). Notably, all those belonged to the high-risk category, with none from the average-risk group adhering to guideline-recommended screening protocols (χ^2^: 22.6, *df*: 1, *P* = 0.00). Furthermore, multiple logistic regression analyses revealed that among high-risk FDRs, male FDRs (OR = 4.4, 95% CI: 1.3–14.8) and those who received recommendations for CRC screening (OR = 17.7, 95% CI: 10.38–24.6) were more inclined to undergo screening (Table 4), and regarding adherence to CRC screening guidelines, IP age under 50 (OR = 14.8, 95% CI: 1.1-27.3), as well as recommendation for screening (OR = 13.31, 95% CI: 1.61-29.8), were two main factors associated with CRC screening according to guideline (Table 5).

**Table 4:** Factors associated with screening colonoscopy in the high-risk group of FDRs of patients with CRC

**Independent variable**	**OR**	**95% CI**	***P*** ** value**
Sex			
Female	1		0.017*
Male	4.4	1.3-14.8	
Ever recommended for screening			
No	1		
Yes	17.7	10.38-24.6	0.00*

Note: FDRs, First-degree relatives; CRC, Colorectal cancer. Analysis method: Multiple logistic regression. *P < 0.05

**Table 5:** Factors associated with screening colonoscopy according to guidelines in the high-risk group of FDRs of patients with CRC

**Independent variable**	**OR**	**95% CI**	***P*** ** value**
IP age (y)			
< 50	14.8	1.1-27.3	0.04*
50-59	5.7	0.45-16.8	0.17
> 60	1		
Ever recommended for screening			
No	1		0.016*
Yes	13.31	1.61-29.8	

Note: FDRs, First-degree relatives; CRC, Colorectal cancer. Analysis method: Multiple logistic regression. *P < 0.05

 Despite physician recommendations and social media promoting early CRC detection, obstacles persisted in the screening process among FDRs of patients. The study identified inadequate awareness of cancer risk and insufficient understanding of the effectiveness of screening tests as significant impediments. 64.4% of FDRs of patients with CRC did not have awareness about the risk of CRC and 56.3% of them who did not undergo colonoscopy did not have knowledge (Table 6). Embarrassment and fear of colonoscopy ranked lowest among the reasons cited for abstaining from the procedure.

**Table 6:** Barriers to all related cancer screening among FDRs of patients with CRC

**Factors**	**N**	**%**
Lack of knowledge	103	64.4
Unawareness of effectiveness	90	56.3
Never see the doctor	78	48.8
The doctor never talks to the patient about CRC screening tests	56	35
Lack of Time	24	15
High cost	10	6.3
Fear of colonoscopy	9	5.6
Fear of experiment	2	1.3
Inaccessibility	2	1.3
Embarrassment of colonoscopy	1	0.6

Note: FDRs, First-degree relatives; CRC, Colorectal cancer.

## Discussion

 In the present study, the CRC screening rate among high-risk FDRs of patients with CRC was relatively elevated compared with that at average risk (64% vs. 36% for high-risk and average-risk, respectively), though this difference was not statistically significant. Notably, adherence to CRC screening guidelines was significantly higher among high-risk FDRs compared with average risks (*P* = 0.00).

 A previous study conducted in Iran has examined CRC screening participation rates based on FDR guidelines, indicating a 50% adherence rate among high-risk groups and 30% among moderate-risk groups.^27^ This study contributes to the limited global data on guideline-conforming CRC screening in FDRs of primary CRC cases, marking a pioneering effort in Iran to assess CRC screening across different risk levels. Internationally, comparison of risk-adjusted screening according to guidelines is challenging due to varying screening modalities and intervals in different countries. For instance, a community-based study conducted in Australia revealed that 45% adhered to recommended CRC screening guidelines for both average and very high risks among individuals aged 55.^8^

 Our findings revealed several factors influencing screening behavior. The likelihood of receiving CRC screening increased with the decrease in IP age but not with the age of the FDRs. Notably, men FDRs with a high risk for CRC exhibited significantly higher SC rates compared to women, and it was also associated with receiving screening recommendations. However, these factors did not exert a notable impact on the participation of average-risk FDRs in CRC screening. In an Iranian study encompassing 1017 FDRs of individuals diagnosed with colon cancer, findings revealed a lack of substantial discrepancy in knowledge and awareness levels between the average-risk and high-risk groups.^27^ However, our investigation demonstrated a higher incidence of colonoscopy uptake in the high-risk FDRs, specifically among those individuals recommended for screenings based on established guidelines (*P* = 0.00).

 In an investigation conducted in Australia involving 405 FDRs of patients, participants were categorized into three groups based on their risk levels: slightly above average risk, moderately increased risk, and potentially high risk. Interestingly, no noticeable distinctions in screening practices were observed among these three risk-stratified groups.^8^ Notably, within the slightly above-average risk group, adherence to screening guidelines was more pronounced among individuals with higher educational attainment and males.^8^ Conversely, our investigation unveiled a distinct trend, wherein screenings demonstrated a male preference only within the high-risk group (*P* = 0.002). Moreover, within the high-risk category of the Australian study, a high prevalence of screening was noted among individuals who were married, lived in metropolitan areas, had siblings, and had received screening information from healthcare professionals.^8^ Interestingly, this screening pattern was not correlated with the sex and age of the Ips.^8^ In contrast to the general trend identified in this study, our study showed that FDRs under 50 years old had higher rates of SC compared with older age groups, although this difference was not statistically significant. However, participation in the screening program was significantly higher among FDRs of IPs diagnosed with CRC under 50, indicating that the younger the age at cancer diagnosis, the greater the importance placed on undergoing SC by their FDRs.

 In contrast to previous studies indicating cost as a hindrance, our study did not find a notable association between cost and CRC screening.^8^ The reason for this might be that this study was conducted in a private clinic, and the patients attending had relatively good financial status compared with studies conducted in governmental centers.^22,25^ Consistent with our research findings, another study conducted in Iran observed no substantial association between health insurance coverage and participation in screening, potentially attributable to insufficient insurance coverage for comprehensive screening procedures in the country.^25^ Additionally, consistent with other studies, our research demonstrated that participation in SC is higher among siblings of patients with CRC compared with other familial relationships (parents and children).^8^ However, marital status, often linked with participation in screening, did not notably influence our results.^8^

 Our findings also indicate that education level and employment status do not significantly correlate with CRC screening. In contrast, another study in eastern Iran found that individuals with higher education and employment engagement exhibit greater awareness of CRC screening.^25^ The discrepancy could stem from study limitations, including a small number of participants.

 In a study conducted in China, a correlation was found between a higher rate of colonoscopy and three questionnaire-identified factors: insurance coverage, a family history of CRC, and a physician’s recommendation for screening.^24^ Similarly, our research indicated that screening was more widespread among FDRs in the high-risk category who received recommendations for CRC screening. Moreover, in agreement with our study, the research in Saudi Arabia showed a gender gap, with women displaying lower adherence to screening than men.^28^ This inconsistency could be potentially explained by the fears and embarrassment women may experience during screening, issues that could be alleviated through the presence of female healthcare professionals.

 In our investigation, while physician recommendations were frequently cited as the primary source of information, numerous FDRs identified a lack of awareness as the primary reason for not undergoing screening. This suggests a gap in comprehension regarding the significance of pre-symptomatic screening. This observation is consistent with prior studies suggesting that although enhanced counseling enhances awareness of CRC screening, it does not necessarily lead to increased uptake of colonoscopy.^11,29^

 In a Turkish study, 36.3% of FDRs were aware of colonoscopy, with physicians being the primary information source; however, only 19.5% proceeded to adhere to CRC screening recommendations.^23^ Despite evidence demonstrating decreased CRC morbidity and mortality among moderate-risk individuals aged over 50, adherence to screening guidelines remains irregular, with reported rates ranging from 18% to 34%.^30^ The likelihood of receiving information about CRC and colonoscopy recommendations was elevated among FDRs registered in private clinics than in teaching/research hospitals and among FDRs with Lynch syndrome compared with others.^23^ The study emphasized the importance of educating physicians treating patients with CRC about the significance of FDR screening and its impact on encouraging patients to adhere to SC guidelines.^30^ The absence of symptoms emerged as a prevalent reason for forgoing screening despite evidence indicating that asymptomatic, low-risk individuals are less inclined to undergo screening.^31^ Therefore, both physician and patient commitment to recommended screening guidelines is crucial for early CRC detection among FDRs of patients with CRC.^30^ Our study also revealed that only 44% of participants were aware of CRC risk, mirroring results from a study conducted in Turkey where low screening participation (11.3%) was predominantly linked to a lack of awareness (81%), emphasizing the imperative for heightened public education and awareness initiatives.^23^ In a retrospective study examining physician knowledge and adherence to CRC screening guidelines, a notable gap was identified in the understanding of screening protocols for high-risk populations among gastroenterologists and oncologists. This highlights the necessity for enhanced training and awareness initiatives, possibly integrated within registry centers, to enhance knowledge and awareness in this area.^23^

 The study implies that increasing awareness about SC among patients with CRC and physicians could boost the motivation of doctors to recommend screening, thereby enhancing participation rates in SC among FDRs of patients with CRC.^23^ Another Iranian study observed that FDRs of patients with CRC participating in a population-based screening program lacked basic knowledge about CRC and screening tests.^27^ Studies conducted in populations with high CRC incidence suggest that heightened risk awareness among FDRs of patients with CRC increases their engagement in screening programs. Despite some participants exhibiting symptoms, a significant portion remained unaware that these could be indicative of CRC. Public awareness regarding CRC screening holds paramount importance, especially in developing nations. Remarkably, most of FDRs in our study were unfamiliar with colonoscopy, with a vast majority unaware of its role in cancer prevention. This highlights the necessity for comprehensive public education and awareness initiatives aimed at enhancing comprehension and engagement in CRC screening, especially among high-risk groups such as FDRs of patients with CRC. While having a family history of CRC did not inherently increase knowledge about CRC and its risk factors, it seemed to elevate awareness regarding the significance of screening tests. A common reason for abstaining from screening in our investigation was the perception of being in good health and consequently deeming screening unnecessary. This highlights the pivotal role of both the public and the healthcare system in comprehending CRC risks and fostering participation in screening.

## Conclusion

 Public awareness regarding CRC screening is pivotal, particularly in developing countries. Our study identified substantial underutilization of screening among high-risk and no-screening colonoscopies adhering to the guidelines in the average-risk FDRs. There is an urgent need to implement effective, systematic interventions at a population level and enhance education for both healthcare providers and patients regarding appropriate risk-based screening for FDRs of patients with CRC. Furthermore, further descriptive investigations are warranted to identify this population’s barriers to CRC screening.
